# Utilization of Fruit Seed-Based Bioactive Compounds for Formulating the Nutraceuticals and Functional Food: A Review

**DOI:** 10.3389/fnut.2022.902554

**Published:** 2022-05-23

**Authors:** Shumyla Allaqaband, Aamir Hussain Dar, Ulpa Patel, Navneet Kumar, Gulzar Ahmad Nayik, Shafat Ahmad Khan, Mohammad Javed Ansari, Nadiyah M. Alabdallah, Pradeep Kumar, Vinay Kumar Pandey, Béla Kovács, Ayaz Mukarram Shaikh

**Affiliations:** ^1^Department of Food Technology, Islamic University of Science and Technology, Awantipora, India; ^2^Department of Processing and Food Engineering, College of Agricultural Engineering and Technology, Anand Agricultural University, Godhra, India; ^3^Department of Food Science and Technology, Govt. Degree College Shopian, Srinagar, India; ^4^Department of Botany, Hindu College Moradabad, Mahatma Jyotiba Phule Rohilkhand University, Bareilly, India; ^5^Department of Biology, College of Science, Imam Abdulrahman Bin Faisal University, Dammam, Saudi Arabia; ^6^Department of Fruit and Vegetable Processing Technology, Institute of Food Science and Technology, Hungarian University of Agriculture and Life Sciences, Budapest, Hungry; ^7^Axis Institute of Technology and Management, Kanpur, India; ^8^Institute of Food Science, University of Debrecen, Debrecen, Hungary

**Keywords:** seeds, pulp, apple, guava, dates, waste management, bioactive compounds

## Abstract

Fruit seeds include a large number of bioactive substances with potential applications in the culinary and pharmaceutical industries, satisfying current demands for natural ingredients, which are generally preferred since they have fewer adverse effects than artificial components. Researchers have long been interested in the functional features, as well as the proximate and mineral compositions, of diverse fruit seeds such as tomato, apple, guava, and dates, among others. Bioactive components such as proteins (bioactive peptides), carotenoids (lycopene), polysaccharides (pectin), phytochemicals (flavonoids), and vitamins (-tocopherol) are abundant in fruit by-products and have significant health benefits, making them a viable alternative for the formulation of a wide range of food products with significant functional and nutraceutical potential. This article discusses the role and activities of bioactive chemicals found in tomato, apple, dates, and guava seeds, which can be used in a variety of food forms to cure a variety of cardiovascular and neurological disorders, as well as act as an antioxidant, anticancer, and antibacterial agent. The extraction of diverse bioactive components from by-products could pave the path for the creation of value-added products from the fruit industry, making it more commercially viable while also reducing environmental pollution caused by by-products from the fruit industry.

## Introduction

Consumer interest in quick meals and healthy eating has sparked the development of functional foods. Functional foods and nutraceuticals, according to the US Dietary and Drug Administration (FDA), are foods or food components that may provide health benefits in addition to their nutritional value and are gaining popularity as a result of ongoing research to uncover qualities and prospective uses of ingredients obtained from food sources. Dietary fiber and antioxidants are two target ingredients for functional meals that are thought to provide health benefits. Mainly fresh produce, high in fiber, and antioxidants can be introduced into the human food chain, resulting in new potential functional foods. The goal of functional food is to improve health or guard against disease. The term “functional food” can also refer to features that have been purposely bred into established edible plants, such as purple or gold potatoes with reduced levels of anthocyanin or carotenoid (1). These foods, which can look like traditional food and be eaten as part of a daily diet, are prepared for functional benefits and aid in reducing the risk of long-term diseases beyond basic nutritional functions (2). They can be prepared for functional benefits and aid in reducing the risk of long-term diseases beyond basic nutritional functions.

Recent research showed that a diet high in antioxidants is linked to a decreased risk of chronic illnesses. Cereals, legumes (wheat, maize, barley, sorghum, nuts, rice, oats, rice, pulses, and beans), oilseeds (flaxseed, rapeseed, olive seeds, and canola), fruits (apple cherry, pear, and different berries) and vegetables are the main sources of dietary polyphenols and many bio-active components which contributes in its functional and nutraceutical potential (2–6). Not only the wholesome fruit but also the waste residue left after processing contains many phenolic compounds and bioactive phytochemicals which are rich in antioxidant, anti-inflammatory, anti-mutagenic and anti-carcinogenic properties (e.g., flavonols, dihydrochalcones, phenolic acids like quercetin glycosides, epicatechin, chlorogenic acid,3-hydroxy-phloridzin and phloridzin and bio-active phytochemicals like tocopherols, sterols, terpenes, polyphenols, and carotene) (7, 8). Fresh fruits and vegetables are typically recommended to minimize loss of nutritional content. They are, however, processed into juices and juice drinks for transportation, shelf-life extension, and other reasons. Pectin, mucilages, and beta-glucan are soluble, whereas lignin, cellulose, and hemicellulose are not digested or absorbed in the small intestine and pass unchanged into the large intestine, immune to enzymatic digestion. Solubility is used to classify fibers as soluble or insoluble. Fiber fortification also improves the sensory quality, shelf life, and structural aspects of dairy goods (9). Vitamins A, C, and E, for example, are antioxidants. The oxidation process begins during processing. Antioxidant action in these vitamins inhibits the oxidation process. When these vitamin-rich foods are taken, they can aid in the treatment of a variety of chronic conditions (10).

Agricultural and industrial wastes are frequently wasted or utilized as low-value by-products during the manufacturing of juices or juice drinks. Several antioxidants might be derived from such residual sources, as previously discussed (11). Earlier literature research reviewed that apple peel is rich in phytochemicals and has been described as a value-added food component for food items to promote excellent health (12, 13). Apple Skin/pulp tissues are composed of cell wall polysaccharides (e.g., pectin, lignin gums, and cellulose and its derivatives) and phenolic compounds (e.g., flavonols, phenolic acids, and dihydrochalcones) and both compounds make up the majority (about 95 percent) of the biomass. Not only these compounds, but apple pomace is also rich in high antioxidants such as chlorogenic acid, epicatechin, its dimer, quercetin glycosides, 3-hydroxy-phloridzin, and Phloridzin. Similarly, tomato peels and seeds that are left over after processing are considered waste and are now used as a by-product. Tomato pomace is produced during the processing of tomatoes, with 60 percent of the seed and 40 percent of the skin (14). Proteins account for 32% of the nutritional value of tomato seeds, total fat (27%), and fiber (18%). Tomato seeds have been utilized as a supplement in animal feed and as a substitute in bread items due to their high protein level (15, 16). Tomato seed oil and Tomato seed extract (TSE) can be used in food preservation because of their thermal stability and antioxidant capabilities (17, 18). Some of the practical applications of tomato waste include the creation of biofuels, enzymes, and medicinal chemicals, as well as the manufacturing of tomato seed oil (19). Gallic acid, trans-cinnamic acid, and quercetin are among the phytochemical substances found in tomato seeds (20). Another important functional food that has been claimed to provide all the possible vitamins and energy sources for centuries is dates. The date palm is a member of the Palmaceae family that grows in dry and semi-arid climates around the world. The tree is extremely significant to the majority of people in those countries, and it is regarded as a high-value commercial fruit crop on the global market. Due to the current uncertainty of global food supplies and an expected future food need, the date palm has the potential to provide a reasonable source of inexpensive food and an energy source. Date trees produce a variety of agricultural by-products, including date seeds, which account for the majority of the by-products (about 6.10–11.47 percent of the whole fruit) that are considered waste. Date seed, on the other hand, can be used to make non-caffeinated coffee or added to animal feed. Date fruit and its by-products contain a high quantity of dietary fiber, palms are also high in minerals such as potassium, iron, and calcium.

Matthaus in 2002 reported that the residue of eight different samples of oilseed contain phenolic compounds with significant antioxidant activity, suggesting that they might be employed as natural antioxidants to preserve fats and oils (21). Later on, it was discovered that the extracts which are high in phenolic compounds and bio-active phytochemicals prevent the risk of cancers (mainly colon cancer), cataracts Parkinson’s disease, and Alzheimer’s disease (22). Phytochemicals are non-nutritive plant compounds with disease-fighting or disease-preventive effects. They are non-essential elements generated mostly by plants to protect them. Similarly, dietary fiber is a form of non-starch polysaccharide that has been linked to better digestive health, a reduction in gastrointestinal disorders, weight management, a lower risk of coronary heart disease, better glycemic control, and a lower risk of some cancers (23).

The relevance of functional foods in the prevention of illnesses has piqued the curiosity of the general public and health experts, and as a result, there has been a growing market for foods that can prevent chronic diseases. The phytochemical contents and general health advantages of numerous fruits and vegetable kinds have been examined under both clinical and laboratory circumstances as a result of a surge in the commercial exploitation of functional foods. Some studies have discovered a significant relationship between healthy fruit and vegetable consumption and a decrease in the risk of fatalities from heart-associated disorders, common malignancies, and other deteriorating illnesses.

The present demographic and health trends are the major causes of the functional food market’s growth. Populations are aging throughout the world. Life expectancy is increasing, as is the proportion of elderly people to the overall population. Obesity is now increasingly recognized as a worldwide problem since its prevalence continues to rise in nations all over the world. In the United States, approximately 62 percent of adult people are overweight, and due to this, 32 percent are suffering from heart disease and the highest rate of fatalities. It has been observed in various epidemiology studies that people who eat fruits, vegetables, and whole-grain regularly are at lowers risk of serious illnesses. Many powerful natural anti-oxidants like ascorbic acids, tocopherols, carotenoids, polyphenols, and lipoic acid, prevent oxidative damage by scavenging free radicals from the body (2, 4).

In this review, we will be discussing the potential health aspects of bioactive compounds extracted from the waste of natural sources which act as nutraceuticals and functional foods in our day-to-day life.

### Role of Bioactive Compounds Obtained From Tomato and Tomato Seeds

The processing of fruits and vegetables generates a large quantity of waste, making its usage difficult and posing serious environmental danger. Although part of the trash is used as animal feed but still huge amount remains and goes like peels and seeds can be used. Tomatoes are one of the most widely grown crops on the planet. Global tomato output was 182 million tonnes in 2018, according to FAOSTAT. Tomatoes are consumed fresh as well as processed into cans, purees, sauces, and other products. By-products including pulp, peels, and seeds are released in substantial amounts (1.5–5%) during this procedure. These goods have been recognized as nutraceutical and bioactive compound sources (24). Seeds are predominantly made up of oil and protein, which distinguishes them from the other parts. Seeds include lycopene, phenols, and other chemicals in fewer concentrations than peel fractions (Table 1) (16). It has been reported that seeds and peels are an abundant source of bioactive compounds including ß-carotene and lycopene, which are proven in several studies to have substantial antioxidant potential (25). Because of their diverse phytochemical composition, tomato seeds are considered a possible natural source of antioxidants, phenolic compounds (PCs), and carotenoids, which help the body to manage a variety of metabolic and physiological processes. Tomato peel and pulp, on the other hand, contain a significant number of bioactive components such as lycopene, flavonoids, β–carotene, and phenolic acids.

**TABLE 1:** Different bioactive compounds reported in seed, skin, and pulp of tomato.

Source	Bioactive compounds	References
Seed	Gallic acid (0.11–6.94 mg/100 g), Ferulic acid (1.67–9.08 mg/100 g), Kaempferol (<0.001–2.01 mg/100 g), Quercetin (<0.001–0.90 mg/100 g), Rutin (0.065–3.53 mg/100 g), Coumaric acid (2.58 mg/100 g), Phloridzin (1.35 mg/100 g), Phloretin (26.72 mg/100 g), Procyanidin B2 (76.62 mg/100 g), Apigenin-7-O-glucoside (0.196 mg/100 g), Kaempferol-3-O-glucoside (415.39 mg/100 g), Luteolin-7-O-glucoside (55.77 g/100 g), Genistein (0.196 mg/100 g), Daidzein (0.02 mg/100 g), Quercitrin (0.003 mg/100 g), Epicatechin (0.026 mg/100 g), Quercetin-3-O-sophoroside (6030 mg/100 g), Isorhamnetin-3-O-gentiobioside (738 mg/100 g), Quercetin-3-O-rutinoside (840 mg/100 g), Quercetin-3-O-β-glucoside (2.61–3.94 mg/100 g), Isorhamnetin (0.34–1.36 mg/100 g), Naringenin (0.16–0.35 mg/100 g), Myricetin (0.34–0.88 mg/100 g), Caffeic acid (0.95–2.19 mg/100 g), Vanillic acid (2.01 mg/100 g), Sinapic acid (1.82–3.56 mg/100 g), Chlorogenic acid (0.05–1.41 mg/100 g), ρ-cumaric acid (0.79–3.96 mg/100 g), Trans-cinnamic acid (0.10–3.01 mg/100 g), Lycopene cis 1 (0.058–0.134 mg/100 g), Lycopene cis 2 (0.106–0.302 mg/100 g), Lycopene cis 3 (0.785–0.943 mg/100 g), Lycopene all trans(0.750–1.207 mg/100 g), Lycopene (1.6–16.70 mg/100 g), β-Carotene (0.093–5.500 mg/100 g), Adenosine (<0.001 μg/100 g), Inosine (42.21 μg/100 g), Guanosine (7.44 μg/100 g)	(30, 36–41)
Skin/peel	Chlorogenic der (33–141.10 mg/kg), p-Coumaric (07.38–26.58 mg/kg), p-Coumaric der (16.70–101.99 mg/kg), Quercetin (5.04–13.68 mg/kg), Rutin (107.06–410.13 mg/kg), Rutin der (36–109.75 mg/kg), Naringenin (73.52–287.62 mg/kg), lycopene (167.43 mg/kg dw) β-carot‘ene (55.20 μg/g dw), lutein (065–1.54 mg/100 g), tocopherols (1.62 g/100 g dw), Caffeic acid-glucoside isomer (0.74 mg/100 g), Caffeic acid (0.55 mg/100 g) Syringic acid (0.547–1.122 mg/100 g), Di-Caffeoylquinic acid (0.812–1.113 mg/100 g), Tri-Caffeoylquinic acid (0.591- 0.662 mg/100 g)	(25, 40, 42)
Pulp	Caffeoyl-hexoside (0.815 μg/g), Caffeoyl-hexoside isomers (0.037–0.184 μg/g), 4-Caffeoylquinic acid (0.16 μg/g), 5-p-Coumaroylquinic acid (0.032 μg/g), Quercetin-3-rutinoside (Rutin) (0.058 μg/g), Synapoyl derivative (0.093 μg/g), Caffeic acid derivative (0.105 μg/g), Lycopene (31.49 mg/100 g DW), β-carotene (10.77–21.46 mg/100 g DW)	(43, 44).

### Supplementation of Foods With Tomato/Tomato Products

Many researchers have looked into the possibilities of reusing tomato seeds, but few have looked into the recycling capacity of tomato peels and pomace. This is due to an extra step in the seed recovery process during industrial processing, and the final results of the protein content in tomato seeds demonstrated their nutritional value while also emphasizing their practical utility (26, 27). Tomato seeds are a promising source of protein, according to this study, with all amino acids exceeding WHO/FAO/UNU (28) standards. Nonetheless, underutilized tomato seeds with high protein quality and adequate nutritional content could be used as a long-term protein supplement in future food compositions (29). Tomato seeds have been utilized as a substitute for vegetable oil, with extraction yields ranging from 10 to 35% of seed dry weight depending on the raw material and extraction process used. As a result, even when considering physical activities (crushing, heating, stirring) were utilized, the pomace retained a significant part of the oil present in the tomato seeds following industrial processing. The oils produced from tomato seeds have high quantities of unsaturated fatty acids including linoleic, palmitic, and oleic acids, as well as physicochemical qualities similar to those found in soybean, sunflower, and cottonseed oils (30). Mironeasa and Codinǎ, (31), combined wheat flour with tomato seed flour, and the results are useful for bakery producers designing new goods to which tomato seed flour can be added, particularly for wheat flours with high wet gluten content and good quality for bread baking (31). Yashini et al. investigated the effects of a partially defatted tomato seed flour (PDTSF) as a fat replacer on the physicochemical and sensory characteristics of millet-based cookies at fat replacement levels of 10, 20, 30, and 40% (w/w). As a result, the findings backed up PDTSF’s use as a whole-food fat replacer in the manufacturing of reduced-fat millet cookies at 10 and 20% fat replacement levels (32). In the production of tarhana, Isik and Yapar (33) used tomato seed meal to partially substitute wheat flour (15, 25, and 35 percent). It was revealed that increasing the amount of tomato seed meal in the formulation improved the insoluble dietary fiber, protein, oil, ash, mineral, total dietary fiber, and total phenolic content of tarhanas, as well as their antioxidant activity (*P* < 0.05) (33).

Persia et al. (34) found that tomato seeds had 8.5% moisture, 25% CP, 20.0% fat, 3.1% ash, 35.1% dietary fiber, 0.12% calcium, 0.58% phosphorus and 3,204 kcal/kg. The moisture content of major staple food like brown rice, wheat, maize, millet, sorghum, and oats is about 14%. Ash content is high in tomato seeds as compared to 1.4 g in brown rice, wheat, and maize, 2.6 g in sorghum, and 2.3 g in oats. Dietary fiber also remains high in tomato seeds as compared to 4, 10.5, 9, and 13.8 g in brown rice, wheat, maize, and sorghum, respectively (35).

PCs, which include flavonoids, are a class of tiny bioactive compounds with a chemical structure that includes at least one aromatic ring and one or more hydroxyl moieties. Food sensory qualities may be modulated by PCs by modifying flavor, taste, color, and astringency. The majority of PCs found in foods are phenolic acids and flavonoids. The phytochemical analysis of aqueous tomato seed extract revealed the presence of fourteen flavonoids, including quercetin, isorhamnetin, and kaempferol derivatives, including 13 for the first time in tomato seeds. The tomato seed extract has a total PC concentration of 20,657 mg/100 g (38). PCs were researched to have potential protective measures by altering numerous cellular signal transduction pathways and stimulating endogenous defensive activities for example anticancer, antibacterial, antimutagenic, anti-inflammatory, anti-neurodegeneration, antiplatelet, and cardioprotective. They are capable of carrying out these functions on their own or in conjunction with other components found in tomato seeds. Also, the bioactivity of dietary phytochemicals exemplifies their potential as functional food and medicinal additives (30).

Several approaches for the valorization of the unused parts of tomato in various sectors have been proposed, different methods were adopted for the extraction and purification of the carotenoid compounds which were further used in the formulation of functional foods, by all the manufacturing companies including pharmaceutical and cosmetic products, rather than using synthetic chemicals. Indeed, the two most important carotenoids found in tomatoes are lycopene and β -carotene. 90% of the Lycopene content is found in the peels and the major content of β-carotene is in the seeds of the tomato. Abundant research has proved that the highest amount of carotenoids is present in the waste of tomatoes be it peels or seeds. It has been reported in the literature that the concentration of lycopene on a dry weight basis or industrial waste level is approximately 377 and 175 g/g whereas the whole tomato contains only 82 g/g (45). Carotenoids, in addition to phenolics, are another type of bioactive substance found in tomato seeds. Carotenoids are pigments that are chemically and functionally varied biomolecules found all over the world. Because of their capacity to scavenge free radicals, carotenoids have huge antioxidant potential (46). Reduced risk of some malignancies, cardiovascular illnesses, age-related macular degeneration, and cataracts are among the many health advantages linked with carotenoid intake (47).

Cardiovascular diseases (CVDs) are now one of the leading causes of morbidity and death in the United States. Obesity and high dangerous cholesterol levels, hypertension, and platelet activation and aggregation are all risk factors for the development of these disorders. Consumption of tomatoes has been linked to cardioprotective benefits (48). Antiplatelet aggregation action is important for atherosclerosis prevention (49). Platelets stick to the endothelial cell lining and aid in the recruitment of leukocytes that define local vascular inflammation in atherosclerosis. The impact of tomato pomace extracts, including seeds, on platelet aggregation, was investigated by Palomo et al. The researchers found that taking 1.0 g of tomato pomace extracts daily for 5 days reduced platelet aggregation and the risk of cardiovascular disease in human participants (48).

While research also studied tomato seed oil it was seen compound phytosterols found in tomato seed oil have a similar structure to cholesterol which helped in the inhibition of cholesterol absorption in the human body. Tomato seed oil lowers cholesterol levels by altering HMG-CoA reductase activity indirectly via sterol regulatory element-binding protein-2 (SREBP-2). The oil extracts also affect the transcription of LDLR (low-density lipoprotein receptors) via SREBP-2, which lowers cholesterol levels. Among carotenoids, lycopene is a valuable component that is useful for human health. Tomato pomace is a by-product of tomato processing that contains peel, seeds, and minor amounts of pulp. Tomato pomace, seed, and peel are frequently reused in various products. Dried tomato wastes can be fed to animals and used as a meat enhancer. Seeds were used in the bakery as well as fermented cereal food. Oils with a high carotenoid content are abundant in the seeds, contributing to the oil’s oxidative stability. Tomato seed oil can also be utilized in biodiesel and other non-food purposes (27).

Tomato Seed Extracts (TSEs) have also shown antibacterial properties i.e., the pharmaceutical and food sectors can use them for food preservation and microbial deterioration. Tomato seeds left after processing were fermented using Bacillus subtilis as culture and were seen to produce hydrolyzates which have high antibacterial and antioxidant potential against Bacillus cereus and Escherichia coli, with growth rates reduced by up to 69.8% and 29.8%, correspondingly (50). The antibacterial characteristics of the hydrolyzate were attributable to bioactive peptides produced during fermentation. Likewise, the antibacterial activities of 10 tomato variety processing wastes were linked to their phenolic and carotenoid levels (25).

Ferreres et al. (38) identified bioactive compounds like Isorhamnetin-3-O-sophoroside, Kaempferol-3-O-sophoroside, and Quercetin-3-O-sophoroside from tomato seeds and found that a considerable cell growth inhibition of >80% was observed at the highest tested concentration (8 mg/mL) with 5980 g/mL IC_50_ value of against rat basophil leukemia (RBL-2H3) cell line. Tomatoside-A bioactive compound was observed in tomato seeds by Li et al. (51) and reported that in human intestinal Caco-2 cells, treatment with 10 μM of this bioactive compound for 3 h resulted in a 46.0 percent decrease in transport of intestinal glucose relative to untreated cells. Ezz et al. (52) identified bioactive compounds lycopene, tocopherols, lutein, and β-carotene from tomato seed oil and reported that before exposure to gamma radiation, an oral dose was given to rats (1 ml/kg at the rate of 3 times per week for a total period of 8 weeks) that protected them from the ionizing radiation effect by reducing oxidative stress, restoring lipid homeostasis, and reducing systemic inflammation. He et al. (53) found that in C57BL/6J mice, a high-dose tomato seed oil (11.8%) diet lowered cholesterol levels. Tomato seed oil (TSO) supplementation’s lipid-lowering impact is likely to be achieved by reducing cholesterol absorption, increasing fatty acid oxidation, enhancing cholesterol efflux, and favorable gut microbiota modification. TSO’s lipid-lowering action is related to the gut microbiota, according to the study.

According to published data, the major fibrous material is present in the tomato pomace, among which 33.6% is the crude fiber, 57.5% is the natural detergent fiber, and 44.5% is acid detergent fibre (ADF) on a dry matter basis, respectively. Besides fiber, it contains crude protein which accounts for an average of 19%, and fat 9.4% on average. Both are reasonably high in tomato pomace. Among the fat, the unsaturated fatty acids account for the highest percentage of 76% with linoleic, oleic, and palmitic acids being the most prevalent fatty acids (54). Being rich in so many nutrients, tomato pomace is viewed as a viable and nutritious supplement to pasture as it contains high nutrients, and especially for its high level of protein, it is widely recommended for animal feed. It’s mostly fed to all poultry (chicken hamsters) and cattle animals (cows, sheep, goats, pigs, rabbits) as a ration nutritious supplement. It has been reported that both the cattle and poultry animal weight increased which led to the increase in quality yield of milk, meat, and egg.

After review, it was found that the peels are high in lycopene, fiber, and phenols, and the seeds are high in crude protein and fat, the waste of tomatoes can be significantly used by industries to develop various by-products or functional foods also the processing manufacture of tomato can benefit from it.

### Role of Bioactive Compounds From Apple Seeds and Pomace

The apple (*Malus domestica*) is one of the most widely grown fruit crops in temperate climates across the world. The fruit is eaten raw or transformed into jam, concentrate, juice, canned goods, and cider. Processing, on the other hand, produces a large quantity of pomace, which accounts for up to 20–35% of the fresh fruit weight and is made up of a mixture of skin/pulp tissue (94.5%), seeds (4.1%), and stems (1.1%) (55, 56). Apple seeds are a good source of proteins (38.85–49.55%) and lipids (20.69–24.32%), with linoleic and oleic acids being the most common amino acids (Table 2) (57). They also contain a significant number of polyphenolics, primarily flavonols, flavan-3-ol, dihydrochalcones (phloridzin), and hydroxycinnamic acid. All these polyphenols are very beneficial to the human body. It helps in lowering the chances of obesity and any type of diabetes mellitus (58–60). The functionality of apple polyphenols is limitless, it has been proven the prevention of bone loss density, stop the growth of cancer cells, enhancing human memory (59).

**TABLE 2:** Different bioactive compounds reported in seed and pulp of apple.

Source	Bioactive compound	References
Seed	Linolenic acid (63.76 g/100 g), Phloridzin (2.96 μg/g), Amygdaline, Oleic acid (34.84 g/100 g) Antioxidant activity [DPPH (0.71 μg TROLOX/g)], Total phenolic acid (1.61 μg GAE/g), Hydroxybenzoic Acid (2.99 mg/g)	(73, 74)
Apple pomace	Malic acid (1.08 g/100 g), Total polyphenol (4620 mg/kg), Quercetin, Phloridzin and Phloretin (3.31 mg/g), Chlorogenic Acid (6.89 mg/g)	(74, 75)
Whole apple	Chlorogenic Acid and Phloridzin (5.90 mg/g)	(74)

Apple seeds are now gaining popularity as a functional food in different formulations and also potential bioactive compounds (59, 61). Apple seeds are being crushed to form Defatted apple seed flour and have been widely researched for use in a variety of food systems. As per the latest research, it has been investigated that the nutritional value of bread supplemented with 20% defatted apple seed cake (DASC) has higher nutrition value like higher protein (15.99%) and insoluble dietary fiber (7.21%) content. Bread treated with 20% DASC also had a greater total phenolic content and antioxidant activity, and also the freshness in the bread stayed for a long time (56).

Apple seeds contain a higher percentage of carbohydrates 71.11% which is higher than all the staples of India. Also, apple seeds contain a good source of 2.74 Nx6.25% crude protein, 7.67% crude fiber greater as compared to the crude fiber of brown rice 4 g and oats 5.5 g. It also contains 15.86% Fats/oil, 2.62% ash, 438.14 g/cal energy, and a moisture content of 66.26% as compared to a moisture content of 14% in major staple food (62).

Furthermore, the functional properties and measure of bioactive compounds or nutritional quality in apple and apple seeds depend upon the breed, size volume shape density, location of cultivation, and others are harvesting, cleaning, separating, sorting, conveying, and grinding (63).

Besides seeds, Thousands of tonnes of apples undergo the processing of fresh cut-slices, juice, sauces, pies or jams, etc., thereby a large quantity of waste is produced which is referred to as apple pomace. This apple pomace is high in simple sugars, phenolics, carbohydrates, proteins, vitamins, minerals, bioactive compounds, terpenoids, and other natural substances (64, 65). The recovered bioactive compounds or nutrients from the apple pomace could be used to improve the composition and activity of different byproducts in food, cosmetics, and medicinal formulations. The apple pomace has broadly gained a lot of attention and many efforts were put by the researchers in recent decades to utilize the by-product for the production of biofuel (ethanol), gasification, and anaerobic digestion (methane generation), pectin and citric acid production, livestock feed (66). It’s critical to find profitable ways to dispose of the generated apple pomace waste or to discover value-added uses that are both economically and environmentally beneficial. Many researchers have also worked on the fermentation of apple pomace to produce bioethanol (67) and biobutanol (68) in biorefineries. Pretreatment is required to liberate the simple sugars present in cellulose and hemicellulose for the fermentation of apple pomace either by acetone-butanol-ethanol (ABE) or alcohol which results in the hydrolyzate rich in sugars like hexsos and pentoses. Due to its bioethanol production capacity and tolerance, Saccharomyces cerevisiae has been preferred for standard carbon sources in alcoholic fermentation, there are also other microorganisms, such as *Scheffersomyces stipitis Kluyveromyces* sp., or *Zymomonas mobilis* are emerging as interesting alternatives for cellulosic biomasses like apple pomace (69, 70). The species *Clostridium beijerinckii* has been effectively used in ABE fermentation from apple pomace.

Paulina et al. (71) experimented on the nutritional properties of apple seed meal (ASM) and its effect on the health of rats. The ASM group received a diet that included 0.24 percent amygdalin from the meal. It was observed that the weight of rats from the ASM group was reduced after 14 days. Also, the ASM diet raised plasma concentrations of high-density lipoprotein cholesterol and water-soluble antioxidant capacity. The amount of thiobarbituric acid-reactive compounds was also reported to be reduced in the liver. The apple seed meal supplementation was also reported to be beneficial for the intestinal tract, antioxidant status, and blood lipid profile of rats.

### Supplementation of Foods With Apple/Apple Products

Puri et al. discovered that adding 5% and 20% DASC of three apple varieties (Golden Delicious, Idared, and Umatovka) to the total amount of wheat flour increased insoluble fiber and protein content significantly in the tested bread samples, particularly in the bread sample supplemented with 20% DASC of umatovka (7.21 and 15.99 percent, respectively). Bread samples added with 20% umatovka had the most insoluble fiber, proteins, total polyphenol content, antioxidant potential, and the least reduction in freshness, whereas bread samples supplemented with 5% Golden Delicious had the most soluble fiber, brightness, and chewiness. Finally, we had a low-energy, high-antioxidant potential, decent texture of an enriched bread with proteins and fiber with an acceptable sensory profile (56). Gunes et al. investigated the kinetics of phloridzin dissolution using defatted apple seeds in a chewing gum system. The results suggested that chewing gum could be a suitable way to distribute phloridzin, and apple seeds, a significant agricultural output, could be used as a test subject (59). The results from research by Rodrígue and Suárez (72) suggest apple seeds from the cider-making industry could be a suitable raw material for food, pharmacological or cosmetic uses (72).

### Role of Bioactive Compounds Found in Seeds of Dates

Phoenix dactylifera otherwise known as Date or Date palm is one of the most popular fruits around the world and is a requisite part of dietary regimes in several cultures. “Dactylifera” has been derived from the Greek word “daktulos” which means a finger, instantiating the form of the fruit (FAO, Date Production Support Programme). Date palm is considered to be cultivated since the 5th millenium B.C., the evidence suggests Southern Mesopotamia as its source (76). It grows very well in arid and semi-arid regions of the world. In the year 2020, ∼9.45 MT of date palm was produced globally. Egypt (1373.57 MT), Saudi Arabia (1122.82 MT), and Iran (1016.61 MT) were the top producers. Initially, its consumption was limited only to Arabic and North African countries but with the advent of the internet, people have realized its worth. Thus, presently it is consumed worldwide mainly due to its nutritional and functional properties. Date fruit is a repository of carbohydrates (glucose, fructose, maltose, mannose, and sucrose constitute more than 80% of dry matter), twenty-three amino acids are found in date proteins including Arginine and Histidine (not many fruits possess that many amino acids), vitamins like ascorbic acid, folic acid, retinol, riboflavin, thiamine which are essential for the human body are present in dates, copious amount of Ca (70.7 mg/100 g), Fe (0.3 – 6.03 mg/100 g), Mg (64.2 mg/100 g), Na (32.9 mg/100 g), P (864 mg/100 g), Zn (0.5 mg/100 g) is present in it along with phenolic compounds and fiber (77–81). Date fruit comprises the epicarp, a fleshy and edible mesocarp also called the pulp, and an endocarp consisting of a seed called a kernel or pit (81). Both, the fruit, and seed characteristics vary depending upon the variety, cultivation, and atmospheric conditions. The weight of the seed can range anywhere between <0.5 to 4 g, breadth 6 to 13 mm, and length 12 to 36 mm. The seed is made of dense cellulose deposit with a small embryo and is oblong and ventrally hooved (FAO, Date Production Support Programme). The pulp to seed ratio can vary from 4.55 to 18.38 depending on different cultivars and stages (82). In most of the varieties, seed amounts to 10–15% of the total weight of the fruit (83). The palm agro and processing industries produce a huge amount of seed as waste. This high wastage rate, mishandling, and scientific nescience may increase the waste up to >30% of production value (84), which produces a huge opportunity of this bio-resource to be utilized into value added products.

Total carbohydrates and crude fiber were substantially higher in date seeds powder at about 52.87 and 27.15%, respectively. Total protein, lipid content, and ash content were also 7.73, 7.90, and 4.35%, respectively. Also, date seeds powder is a good source of the total, insoluble, and soluble dietary fibers (67.56, 46.38, and 21.18%), respectively (85). Salama et al. (86) found carbohydrates in tomato seeds as 63.92%, which is considerably more than carbohydrates available in staple food like wheat 61.6 g, maize 60.9 g, and sorghum 57.4 g (35). The ash content (1.78%) is also higher than the ash content of 1.4 g in brown rice, wheat, and maize, 2.6 g in sorghum, and 2.3 g in oats. The moisture content of date seed powder is about 4.05%, which is less as compared to 14% in major food like brown rice, wheat, maize, millet, sorghum, and oats.

Carotenoids (tetraterpenoids) are present in a plentiful amount in date seeds (Table 3). These provide health benefits by preventing the occurrence of chronic diseases. Habib and Ibrahim performed carotene analysis in more than one and a half dozen date varieties of UAE (88). They found that β-carotene (3142 μg/kg) is the primary form of carotenoid in date seed oil followed by lutein (1599 μg/kg) other carotenoids were present in a very scarce amount of zeaxanthin (10.8 μg/kg), lycopene (19.5 μg/kg) and β-cryptoxanthin (20.4 μg/kg).

**TABLE 3:** Different bioactive compounds reported in seed and whole fruit of the date.

Source	Bioactive compound	References
Seed	lignin (27.34%), cellulose (20.63%), hemicellulose (13.49%) Protocatechuic (7.9 mg/100 g), Caffeoylshikimic (28.3 mg/100 g)	(73, 87)
Whole fruit	4-Hydroxybenzoic (0.16 mg/100 g), Gallic acid (0.16 mg/100 g), Protocatechuic (2.27 mg/100 g), Syringic (2.45 mg/100 g), Vanillic (1.76 mg/100 g), Caffeic (3.37 mg/100 g), Ferulic (9.62 mg/100 g), o-Coumaric (0.50 mg/100 g), p-Coumaric (2.89 mg/100 g)	(87)

Adeosun et al. showed that date seeds contain 102.27 (mg/100 g) alkaloids, anthraquinones, saponins, tannins, and terpenoids. Their study showed that these seeds lower the occurrence of some CV conditions and cancer as well as strengthen the immune system. They concluded that date seed is rich in flavonoids (polyphenol antioxidants) which are responsible for a significant free radical scavenging effect (89). Bouhlali et al. studied three varieties (Majhoul, Boufgous, and Bousthammi) of Moroccan dates and they found that their seeds contain very high flavonoid content (1224–1844 mg RE/100 g) (90). Habib and Ibrahim found that the major group of compounds in flavonoids were flavan-3-ols constituting about 99% of total polyphenols which were distributed as catechin (3.38 619 g/kg) and epicatechin (46.8 g/kg) (88).

Phytosterols are phytochemicals that are soluble in fat and have a structure similar to cholesterol. Phytosterols are efficacious against hormone-related diseases and health conditions, and they have been used against the same for ages. They are antidiabetic, antioxidant, and anti-inflammatory (91). They are present in date seed oil in abundance. The predominant fraction is Δ-sitosterol (76%). Δ5,24-stigmastadienol, Δ5-avenasterol and campesterol are also present in significant amounts but Δ7-avenasterol, cholesterol and stigmasterol are present in less amount (92).

Polyphenols are one of the largest groups of secondary metabolites. Habib and Ibrahim, reported caffeic acid, coumaric acid, and protocatechuic acid in the date seed of the khalas cultivar (88). Al-Farsi and Lee analyzed the seed of the Mabseeli date cultivar and found nine phenolic acids (78). Four were derivates of vanillic and benzoic acid, p-hydroxybenzoic, o-coumaric acid and protocatechuic acid were majorly present. Five derivatives were of cinnamic acid (caffeic acid, ferulic acid, m-coumaric acid, p-coumaric acid, and o-coumaric acid).

Vitamin E (tocopherols and tocotrienols) are also present in date seeds. It is a lipid-soluble vitamin and cannot be synthesized by the human body. It acts as an antioxidant and scavenges the free radicals. Thus it protects the body from aging, arthritis, atherosclerosis, cancer, and cataract. Vitamin E also helps in reducing prostaglandin production (93). Nehdi et al. reported Υ-tocopherol (10.30 mg/100 g) and Υ-tocotrienol (4.63 mg/100 g) as the major tocopherols in seeds of Tunisian dates, their study also reported the significant presence of α-tocopherol (which has higher oxygen and light stability than tocopherol) (92).

Various *in vitro* studies demonstrate that date seeds have anti-bacterial properties (inhibit the growth of gram-positive and gram-negative bacteria like Bacillus cereus, E. coli, Salmonella typhi, and *Staphylococcus aureus*) (94), antifungal activities (against *Fusarium oxysporum*; boost the effect of amphotericin B), antioxidant activity and antiviral activity (77, 90, 94–96).

In the Vivo studies, the anti-cancer activities of date seeds were recently demonstrated by Hilary et al. (97). Zhang et al. also demonstrated similar results to Inhibit cell proliferation in gastric, colon, breast, and lung tumor lines (96).

Date seed extract was given to male rats at a dose of 2 ml/kg orally for 2 months in an experiment conducted by Orabi and Shawaky (62) who reported that daily administration of date palm pits orally resulted in considerable increases in hemoglobin, MCHC (Mean corpuscular hemoglobin concentration) and MCH (mean corpuscular hemoglobin). Malondialdehyde levels in testicular tissue of male albino rats were reduced after daily oral treatment of seeds extract. Salama et al. (86) studied date seed powder (DSP) nanoparticles and infused them as a nutraceutical source in Wistar rats. They experimented on twenty-five rats distributed among 5 groups; (control), on HFD (High-fat diet), HFD and DSP NP1(Nanoparticles) 1:5, on HFD and DSP NP2 2:3, and HFD and DSP infusion 1 g/kg/day). The total cholesterol levels were considerably lowered in all DSP treatment groups. CRP (C-reactive protein) and SAA (Serum amyloid-alpha) levels were significantly reduced by both DSP NPs.

### Role of Bioactive Compounds Obtained From Guava Seeds

Guava fruits are often processed for different products, such as juice, nectar, jelly, squash, wine, confectionery, and jam, resulting in so-called guava processing residues including peel, pulp, and seeds (98).

Guava fruit is obtained from tropical and subtropical fruit crop *Psidium guajava* L. It is an evergreen shrub or small tree, belonging to the *Myrtaceae* family. Archeological evidence suggests that guava was used in Peru around 800 BCE and very likely it was domesticated in Peru. It is believed that the Portuguese and Spaniards transmitted it to other parts of the world. *Psidium guajava* is the most important fruit of the genus Psidium (which contains about 150 species) (99). Guava fruit is also known as the “tropical apple” and “the fruit of the poor guy.” Guava fruit is strong and musky when ripe and generally round but can also be ovoid and pear-shaped. It is 5–10 cm long, with light yellow skin with pink spots scattered all around. Under the skin is a layer of granular pulp (3–12.5 mm thickness). The flesh is yellowish-white, light/dark pink, sweet, mildly acidic, acidic. The pulp is filled with hard yellowish seeds (some cultivars have soft and chewable seeds, but it is very rare). The actual seed count of the fruit ranges from 112 to 535 in number but some varieties are seedless as well (PU, ONLINE EDU).

The seeds are discarded in the food and beverage processing industry, but the seeds are of utmost importance because they are highly nutritious and contain several bioactive compounds in generous amounts. (Uchôa-thomaz et al. (100)) analyzed guava seeds of the paluma cultivar (100). Seeds are 6–12% of the fruit (101). They found that guava seeds have reduced calorie content (182 kcal/100 g), very high dietary fiber (63.94 g/100 g), (13.8 mg/100 g) iron, (3.31 mg/100 g) zinc, and protein (11.19 g/100 g). Their results showed that unsaturated fatty acids were primarily present (87.06%), especially linoleic acid and oleic acid. Bioactive compounds were also significantly present like ascorbic acid (87.44 mg/100 g), insoluble dietary fiber (63.55 g/100 g), and total carotenoids (1.25 mg/100 g) (Table 4) (100). (Prasad and Azeemoddin, (102)) showed that guava seeds contain more than 87% unsaturated fatty acids compared to only 11.8% saturated fatty acids. Studies from (104) also confirmed that guava seeds have a good amount of important minerals (102).

**TABLE 4:** Different bioactive compounds reported in seed, pulp, and peel of guava.

Source	Bioactive compound	References
Seed	Vitamin C (87.44 ± 1.70 mg/100 g), Carotenoids Totals (1.25 ± 0.14 mg/100 g), Soluble Dietary Fiber (0.39 ± 0.02 g/100 g), Insoluble Dietary Fiber (63.55 ± 0.12 g/100 g), Linoleic acid (75.25%), Oleic acid (11.40%), Palmitic acid (6.6%), 2-Chloroethyl linoleate (44.53%), 2-Pentanone 4-hydroxy-4-methyl (23.56%), n-Hexadecanoic acid (9.85%), Tocopherol (α-tocopherol) (654 ppm), Caretonoids (β-carotene) (19.24 ppm), Phytosterol (stigmasterol, campesterol) (420 mg/100 g), Vanillin (9.6 mg/100 g), Vanillic acid (3.9 mg/100 g), Cinnamic acid (2.4 mg/100 g), Cinnamaladehyde (9.4 mg/100 g), β-sitisterol (1048.9 mg/100 g), γ-tocopherol (82.6 mg/100 g),	(73, 100)
Pulp	Gallic acid (79.80 ± 1.03%) Catechin (20.20 ± 1.03%) Carotenoids (50.10 ± 0.13 μg lycopene/g) L-ascorbic acid (57.53 ± 0.72%)	(103)
Peel	Anthocyanin (121.85 Eq. mg of cyanidin-3-glycoside/100 g), Catechin, β-carotene	(73)

Uchôa-Thomaz et al. (100) reported the moisture of guava seeds is about 6.68 g/100 g. The ash content, total lipids, protein, carbohydrate, pectin, fructose, starch, total dietary fiber, and total calories are 1.18 g/100 g, 13.93 g/100 g, 11.19 g/100 g, 3.08 g/100 g, 0.58 g/100 g, 0.29 g/100 g, 0.17 g/100 g, 63.94 g/100 g and 182 Kcal/100 g respectively. The carbohydrate content in guava seeds is lower than carbohydrate in brown rice 71.1 g, wheat 61.6 g, maize 60.9 g and sorghum 57.4 g, and oats 63 g. The ash content is also less than brown rice, wheat, and maize, 2.6 g in sorghum and 2.3 g in oats. The protein content in guava seeds is also less in comparison to brown rice, wheat, maize, millet, sorghum, and oats, respectively. Dietary fiber in guava seeds is found high as compared to 4.0, 10.5, 9, 37, and 13.8 g in brown rice, wheat, maize, millet, and sorghum, respectively (35).

Phenolic compounds have received much attention due to their free radical scavenging and antioxidant ability which have a direct beneficial impact on human health (105, 106). Total phenolic content is 973.80 ± 42.06 (mg/100 g) in guava seeds (101). da Silva and Jorge, (107) showed that p-Coumaric acid (48.40 mg/1000 g), Salicylic acid (36.30 mg/1000 g), and Quercetin (13.87 mg/1000 g) were primarily present as phenolic acids in guava seeds. (da Silva and Jorge, (107)) reported total phytosterols (4654.7 mg/kg) as β-Sitosterol (4376.4 mg/kg) and Stigmastanol (266.2 mg/kg) (107).

Flavonoids are naturally occurring polyphenols found in fruits, vegetables, leaves, seeds, flowers, and grains (108). el Anany, (101) showed that guava seeds contain 290.30 ± 16.03 (mg/100 g) flavonoids (101). Flavonoids have aided in coronary heart diseases and have anticancer activities while some have the potential for antihuman immunodeficiency virus functions (109).

Total tocopherols estimated by (da Silva and Jorge, (107)) was 146.40 (mg/kg), Υ-Tocopherol (95.40 mg/kg) and α-Tocopherol (47.03 mg/kg) being predominant over β-Tocopherol (3.97 mg/kg). Meanwhile, total carotenoids were 11.94 (μg β-carotene/g) (107). The antioxidant activity of guava seeds was 63.74 to 68.34% as reported by Anany (101). The antioxidant activity was measured using DPPH radical scavenging method. El Anany (101) reported the reducing power of guava seeds to be 0.567. Reducing power is used to determine the ability of antioxidants to donate an electron (101, 110).

The metabolic profile in Wistar rats was studied for evaluating the effect of guava seed consumption by Flávia et al. (111). By feeding guava seeds to the rats, there was a significant reduction in glycemia, triacylglycerides, and total cholesterol, as well as an increase in high-density lipoprotein cholesterol levels. Also, the enzymes alanine aminotransferase (ALT), aminotransferase (AST), and alanine were likewise lowered in concentration. Hassan et al. (112) performed *in vivo* experiment on albino rats for identifying the potential of polyphenols extracted from guava seeds. Two polyphenol extract doses of 200 and 250 mL/kg/bw were given. A significant decrease in triglyceride and blood glucose was observed (*p* < 0.05) in comparison to control and reported in inhibition of α-amylase and glucose transport. Furthermore, increase in red and white blood cells (RBC & WBC), packed cell and mean corpuscular volume (PCV & MCV), and platelets levels significantly.

### Supplementation of Foods With Guava/Guava Products

Aly (113) did a study in which biscuits were made using guava seeds powder at various percentages of 5, 10, and 15%. Various biscuit samples were evaluated on chemical, rheological, sensory, and microbiological levels. Guava seeds were included to boost the functionality of biscuits prepared from it. Guava seed powder added to biscuit dough improved dough stability, elasticity, and energy values while lowering water absorption and softening. Guava seed powder was discovered to increase the nutritional and rheological characteristics of wheat flour-based biscuits (113). Serna-cock et al. investigated the use of guava seed flour as a nitrogen source in alcoholic fermentation, finding that it increased product output and substrate conversion. These findings suggest that guava seed flour could be used as a low-cost, long-term supply of nitrogen for alcoholic fermentation (114). Several studies looked into using guava seed and pomace powder in cupcakes. Cake formulations having 5 percent to 10% flour generated better results, however, formulations containing more than 10% flour had negative effects on cupcake qualities. Weight, volume, and specific volume increased as the ratios of guava by-products decreased (115). According to Khalifa et al. the cake with a high ratio of guava seed flour had higher ratios than the cake with guava peel; this could be because the cake with a high ratio of guava seed flour has higher protein and fat contents. When compared to the control, water-absorbing matrixes, such as soluble fibers in by-product flours, increase WHC and cake volume. Increasing the quantity of flour made from by-products, on the other hand, may have a significant impact on dough gas retention, while fat may help to increase it (116).

### Components of Fruit Seed Oil

Water, proteins, carbs, lipids, ashes, fibers, and minerals make up fruit seeds. Genetic, environmental, and agronomic factors influence the chemical makeup of seeds, which differs by species (botanical origin). Lipids account for a large component of the chemical makeup of fruit seeds. Fruit seeds have oil content ranging from 1.1–1.9 percent (in avocado seeds) to 78.9 percent (in lemon seeds), with the oil content in most fruit seeds being close to one-third of their dry weight. Cranberry, strawberry, and prickly pear seeds had low oil content (below 10%), while apricot, melon, olive, orange, peach, and watermelon seeds had high oil content (about 50% or greater). Considerable differences in the lipid content were observed among studies for the same kind of fruit seeds, mostly explained by the fruit variety, as in grape (117, 118), mango, orange, passion fruit, pear, pomegranate, and plum seeds (119).

#### Triacylglycerols

The fatty acids profile (FA) is useful for determining the stability and nutritional quality of vegetable oils, and the triacylglycerols (TAG) profile is useful for determining their physical and functional qualities. The amount of TAG in an oil impacts its qualities, such as crystallization and melting behavior. A higher crystallization enthalpy is caused by a higher degree of structured fatty acid (SFA) and a uniform FA distribution within the TAG backbone, for example (120).

#### Sterols

Phytosterols are present in seed oils in small amounts mostly in the free form (>97% of total sterols), as observed for grape seeds (117). High variability in the sterol content of fruit seed oils has been assigned to the fruit cultivar and the extraction conditions. Significant differences in the total sterol content and the sterol, profile were observed for seed oils of apple, apricot, and grape (118) from different varieties. A negative correlation was observed with the oil yield, suggesting that a higher oil yield results in a lower concentration of total sterols.

### Health Benefits of Fruit Seed Oils

Phytonutrients and phytochemicals, natural chemical substances that participate in numerous biological processes and contribute to an improvement in health status, or prevent or ameliorate illness conditions, contribute to the nutritional and health value-added of fruit seeds and their oils. Other chemicals such as phenolics, tocopherols, and carotenoids, as well as lipids such as FA, sterols, or polar lipids, have been linked to similar positive benefits. Antioxidant, antiproliferative, anti-inflammatory, and anti-diabetic properties have all been established in fruit seed oils. *in vitro* studies and natural extracts with a high chemical variety of chemicals have been used in most of the published work to demonstrate these actions. As a result, establishing a putative structure-function link is difficult. The favorable biological effects are thought to result from a synergistic impact between various components of lipid extracts or oils in most circumstances (119).

### Therapeutic Potentials of Fruit Seed-Based Bioactive Compounds

Chia, hemp, sesame, pumpkin, sunflower, mustard, nigella, guava, papaya, mangosteen, honeydew, pomegranate, fennel, fenugreek, cumin, sweet orange, cucumber, jackfruit, mango, melons, avocado, and many other tropical fruits with edible seeds grow abundantly in India. These products, such as the seed kernel, which accounts for 10–35 percent of the total weight, have a significant nutritional value as well as medicinal potential. The nutritional, pharmacological, therapeutic uses, functional qualities, and bioactive contents of several fruits’ seeds are investigated for their functions and applications as food sources and bioactive phytochemical constituents. The seeds contain essential bioactive components such as alkaloids, carotenoids, flavonoids, glycosides, saponins, terpenoids, tannins, steroids, and polyphenolic compounds, which have anti-inflammatory, antioxidant, anti-cancer, anti-diabetic, anti-hyperlipidemic, anti-obesity, neurological disorders, cardiovascular, skin diseases, and chronic diseases properties. They offer exceptional physicochemical qualities as well as high carbohydrate, lipid, protein, vitamin, and mineral content. However, significant research may be conducted to establish the usefulness of nutritional and bioactive components in various seed kinds, as well as their bioavailability and potency. To determine the medicinal and functional potentials of these fruit seeds, extensive research on the seed components can be conducted (121).

## Conclusion

From this study, it could be concluded that the waste of various fruits mostly seeds can be utilized and has great potential as a functional food despite using any artificial source to enrich our food products which not only will degrade human health but also will be a source of various diseases. The utilization of fruit waste will not only minimize environmental pollution but also is beneficial for human health in every aspect and prevention of various diseases as discussed above. Within the circular economy principles of production and usage of natural resources, the potential use of fruit seeds for phytochemical recovery is critical. Bioactive-rich extracts can be used in the pharmaceutical or food industries, or both in some situations. The effects of fruit seed components and oils on human health, such as cancer, inflammatory processes, immunity, diabetes, and other disease conditions, have been rarely investigated *in vivo*, although the data for some fruit seeds are promising. Lipidomics can be used to further examine the structure-activity relationship in regards to bioactive lipids to find the bioactive substance (s). These findings, combined with the United Nations’ Sustainable Development Goals to improve resource efficiency, reduce waste, and mainstream sustainability practices in the exploitation of agro-food industrial by-products, will undoubtedly raise the value of fruit seeds as functional foods and a source of several compounds of a great commercial interest with various potential applications in a variety of industries. Future research should concentrate on the development of novel extraction techniques as well as the biological effects of bioactive-rich extracts *in vitro* and *in vivo*.

## Author Contributions

PK, UP, and AD contributed to the conception and design of the study. SA, GN, NK, VP, and SK wrote the first draft of the manuscript. SA, NA, AS, and BK wrote the sections of the manuscript. MA, AD, and GN have revised the manuscript critically. All authors contributed to the article and approved the submitted version.

## Conflict of Interest

The authors declare that the research was conducted in the absence of any commercial or financial relationships that could be construed as a potential conflict of interest.

## Publisher’s Note

All claims expressed in this article are solely those of the authors and do not necessarily represent those of their affiliated organizations, or those of the publisher, the editors and the reviewers. Any product that may be evaluated in this article, or claim that may be made by its manufacturer, is not guaranteed or endorsed by the publisher.
